# Impact of the food grade heat-killed probiotic and postbiotic oral lozenges in oral hygiene

**DOI:** 10.18632/aging.203923

**Published:** 2022-03-02

**Authors:** Chiao-Wen Lin, Yi-Tzu Chen, Hsieh-Hsun Ho, Yi-Wei Kuo, Wen-Yang Lin, Jui-Fen Chen, Jia-Hung Lin, Cheng-Ruei Liu, Chi-Huei Lin, Yao-Tsung Yeh, Ching-Wei Chen, Yu-Fen Huang, Chen-Hung Hsu, Pei-Shan Hsieh, Shun-Fa Yang

**Affiliations:** 1 Institute of Oral Sciences, Chung Shan Medical University, Taichung, Taiwan; 2 Department of Dentistry, Chung Shan Medical University Hospital, Taichung, Taiwan; 3 School of Dentistry, Chung Shan Medical University, Taichung, Taiwan; 4 Research and Development Department, Bioflag Biotech Co., Ltd., Tainan, Taiwan; 5 Aging and Disease Prevention Research Center, Fooyin University, Kaohsiung, Taiwan; 6 Institute of Medicine, Chung Shan Medical University, Taichung, Taiwan; 7 Department of Medical Research, Chung Shan Medical University Hospital, Taichung, Taiwan

**Keywords:** oral cavity, probiotics, oral diseases, oral microbiota, immunity

## Abstract

The oral cavity plays a crucial role in food digestion and immune protection. Thus, maintaining oral health is necessary. Postbiotic and heat-killed probiotic cells have shown increased antibacterial potential with stable viability compared with live strains. However, clinical evidence regarding their effect on oral health is insufficient. Therefore, in this study, we tested postbiotic lozenges of *Lactobacillus salivarius* subsp. *salicinius* AP-32, *L. paracasei* ET-66, and *L. plantarum* LPL28 and heat-killed probiotic lozenges of *L. salivarius* subsp. *salicinius* AP-32 and *L. paracasei* ET-66 for their effect on oral health. In total, 75 healthy individuals were blindly and randomly divided into placebo, postbiotic lozenge, and heat-killed probiotic lozenge groups and were administered the respective lozenge type for 4 weeks. Postbiotic and heat-killed probiotic lozenge groups demonstrated antibacterial activities with a considerable increase in *L. salivarius* in their oral cavity. Furthermore, their salivary immunoglobulin A, *Lactobacillus*, and *Bifidobacterium* increased. Subjective questionnaires completed by the participants indicated that participants in both the experimental groups developed better oral health and intestinal conditions than those in the placebo group. Overall, our study revealed that a food additive in the form of an oral postbiotic or heat-killed probiotic lozenge may effectively enhance oral immunity, inhibit the growth of oral pathogens, and increase the numbers of beneficial oral microbiota.

## INTRODUCTION

The mucosal immune system of the oropharyngeal cavity, which is the opening gate for digestive and respiratory systems, plays a crucial role in preventing pathogen entry into the human body [1]. The oral cavity is a suitable environment for microbial colonization; 392 taxa with approximately 700 species and 1500 microbial genomes have been identified as residing therein. Firmicutes, Actinobacteria, Proteobacteria, Fusobacteria, Bacteroidetes, and Spirochaetes constitute 96% of all oral bacterial phyla [2, 3]. Additionally, 85 fungal genera are found in the oral cavity [4]. Oral microbes colonize the oral cavity as a biofilm, which regulates oral homeostasis, oral immunity, food digestion, detoxification, inflammatory processes and is involved in disease prevention [5, 6].

A healthy oral environment consists of multiple symbiotic microbiota. Dewhirst et al. demonstrated major oral microbial phylum were Firmicutes (36.7%), Bacteroidetes (17.3%), Proteobacteria (17.1%), Actinobacteria (11.6%), Spirochaetes (7.9%), Fusobacteria (5.2%) [7]. However, poor oral hygiene may cause oral microbiota dysbiosis, which leads to dental bacterial plaque, gingivitis, and periodontitis [8]. In periodontitis, an outgrowth of pathogenic bacteria occurs, among which *Actinobacillus actinomycetemcomitans*, *Porphyromonas gingivalis*, and *Fusobacterium nucleatum* have been reported to be highly related to periodontal disease pathogenesis [9, 10]. The World Health Organization (WHO) estimated that approximately 20%–50% of the global population has periodontitis [11]. Patients with periodontitis are at high risk of developing stroke, peripheral artery disease, and coronary heart disease [12]. The WHO suggested that strategies for periodontitis prevention include practicing oral hygiene, having a healthy diet, using fluoride and antimicrobial agents, and smoking cessation [13]. Studies have shown that live probiotic strains inhibit oral pathogens and are the rationale for periodontal treatment; they are similar to antibiotics but without the major concern of antimicrobial resistance [14]. However, manufacturing viable probiotics is not feasible owing to challenges regarding preservation and viability stabilization [15]. Metabolites of viable probiotic strains (postbiotic) such as 10-Hydroxy-cis-12-octadecenoic acid and heat-inactivated probiotics have shown the potential to alleviate the disruption of the gingival epithelial barrier caused by periodontitis [16].

In 2019, the panel of International Scientific Association for Probiotics and Prebiotics (ISAPP) defined the term ‘postbiotics’ as a preparation of inanimate microorganisms and/or their components that confers a health benefit on the host [17]. Postbiotics are often considered as metabolites secreted by probiotic strains during fermentation and consist of microbial cell fractions, polypeptides, peptidoglycan-derived muropeptides, bacteriocins, peroxides, pili-type structures, short-chain fatty acids, teichoic acid, folate, vitamins, lactic acid, and extracellular polysaccharides. Probiotic components benefit human health, provide nutritional support, competitively inhibit pathogenic bacteria, and regulate the immune system [18, 19]. In addition, the fermentation products of lactic acid bacteria (postbiotics) have a unique flavor and beneficial nutrients and are therefore widely used in the food industry [20]. An *in vitro* study demonstrated that *Lactobacilli* postbiotics reduce colonization levels of *A. actinomycetemcomitans*, which are related to periodontitis [21]. However, clinical evidence proving that postbiotics reduce oral pathogenic bacteria and improve oral health is lacking. Additionally, our previous study revealed that certain heat-killed probiotics, including *L. salivarius* subsp. *salicinius* AP-32 and *L. paracasei* ET-66, effectively limit the growth of oral pathogenic bacteria *in vitro* [22]. Current clinical data indicate that oral lozenges made of viable strains, including *L. salivarius* subsp. *salicinius* AP-32, *L. paracasei* ET-66, and *L. plantarum* LPL28, can increase beneficial microbiota in the oral cavity, reduce the colonization of periodontitis-related bacteria, and increase the levels of salivary immunoglobulin A (IgA) [23].

Based on previous *in-vitro* screening of viable probiotic strains for improving oral health, we further investigated whether heat-killed probiotic (ET-66 and AP-32), and postbiotic lozenges (LPL28, ET-66, and AP-32) modulate oral microbiota, inhibit oral infectious pathogens, and change salivary IgA levels [22, 23]. The results can be applied in the production of supplementary foods for clinical oral health care in future.

## RESULTS

### Postbiotics of AP-32, ET-66, and LPL28 strains showed effective bactericidal effects on oral pathogens *S. mutans*, *P. gingivalis*, *F. nucleatum* subsp. *polymorphum*, and *A. actinomycetemcomitans*

**Figure 1:**
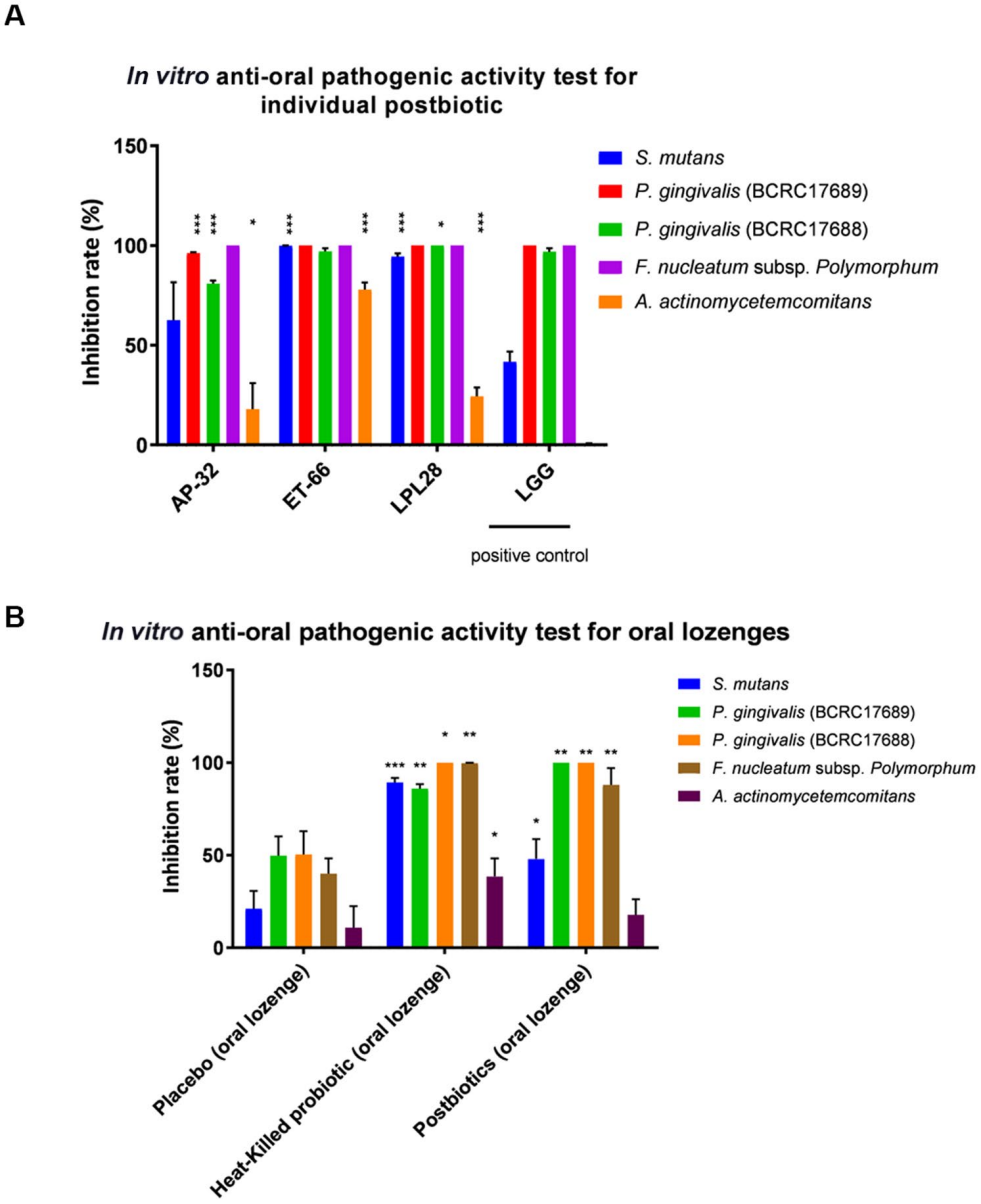
*In vitro* test for determining the antipathogenic activity of (**A**) individual postbiotic and (**B**) probiotic oral lozenges against oral pathogens. (**A**) Postbiotics of AP-32, ET-66, and LPL28 showed strong antibacterial activities compared with the positive control of LGG postbiotic. (**B**) Heat-killed AP-32 and ET-66 were used as inactivated probiotics, whereas metabolites of AP-32, ET-66, and LPL28 were used as postbiotics. *p < 0.05, **p < 0.01, and ***p < 0.001 compared with the positive control group (LGG postbiotic) or the placebo group (without the postbiotic). Data are presented as mean ± SD.

The experimental design was revealed in supplementary data (Supplementary Figure 1). First, we generated the fermentation products of AP-32, ET-66, and LPL28 as postbiotic oral lozenge, and examined its antipathogenic activity against oral pathogens (Figure 1). Compared with the postbiotic of a commercially available strain (LGG), the fermentation products (postbiotics) of AP-32, ET-66, and LPL28 strains had stronger bactericidal effects on oral pathogens, particularly *S. mutans* and *A. actinomycetemcomitans* (Figure 1A). The inhibition rates of *S. mutans* were significantly higher with the use of the postbiotics of AP-32 (62.6%), ET-66 (99.86%, p < 0.001), and LPL28 (94.35%, p < 0.001) than with the use of LGG postbiotic (41.64%). In addition, the inhibition rates of *A. actinomycetemcomitans* were significantly higher with the use of the postbiotics of AP-32 (17.92%, p < 0.05), ET-66 (77.86%, p < 0.001), and LPL28 (24.28%, p < 0.001) than with the use of LGG postbiotic (13.69%). All postbiotics effectively inhibited periodontal pathogens *P. gingivalis* BCRC 17689, *P. gingivalis* BCRC 17688, and *F. nucleatum* subsp. *polymorphum* (Figure 1A). LPL28 postbiotics had a higher inhibition rate of *P. gingivalis* BCRC 17688 (100%, p < 0.05*) than did LGG postbiotic (96.83%).

### Postbiotic and heat-killed probiotic lozenges were effective, demonstrating *in vitro* bactericidal ability against oral pathogens

We prepared two oral lozenges, one with the postbiotics of AP-32, ET-66, and LPL28 and the other with heat-killed AP-32 and ET-66 probiotics for an *in vitro* bactericidal test before launching clinical trials (Figure 1B). The postbiotic oral lozenge group showed a significant increase in the inhibition rates of *S. mutans*, *P. gingivalis* (BCRC 17689), *P. gingivalis* (BCRC 17688), *F. nucleatum*, and *A. actinomycetemcomitans*, namely increases of 47.97% (p < 0.05, placebo = 21.02%), 100% (p < 0.01, placebo = 49.74%), 100% (p < 0.01, placebo = 50.38%), and 87.88% (p < 0.01, placebo = 39.96%), respectively, compared with the placebo group. The inhibition rate of *A. actinomycetemcomitans* in the experimental group slightly increased without a significant difference compared with the placebo group (oral lozenge: 17.78%; placebo: 10.85%). The heat-killed probiotic group significantly inhibited *S. mutans*, *P. gingivalis* (BCRC 17689), *P. gingivalis* (BCRC 17688), *F. nucleatum*, and *A. actinomycetemcomitans* by 89.32% (p < 0.001), 85.91% (p < 0.01), 95.32% (p < 0.05), 91.75% (p < 0.01), and 48.46% (p < 0.05), respectively.

### Postbiotic and heat-killed probiotic lozenges effectively reduced pathogenic colonies in the saliva samples of participants

**Figure 2:**
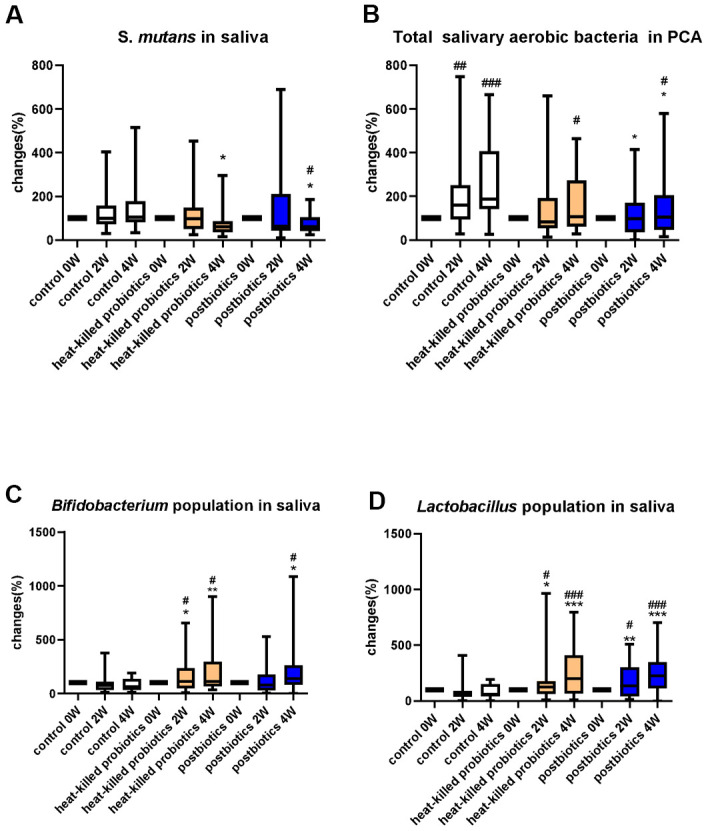
**Microbial change (%) in saliva samples.** Change (%) in the population of (**A**) *S. mutans*, (**B**) total bacteria, (**C**) *Bifidobacterium*, and (**D**) *Lactobacillus* in participants’ saliva at 0, 2, and 4 weeks of oral lozenge intake. The oral lozenges contained postbiotics or heat-killed cells. Participants in the control group consumed placebo lozenges without the postbiotic content (*p < 0.05, **p < 0.01, and ***p < 0.001 compared with the control group; #p < 0.05, ##p < 0.01, and ###p < 0.001 in reference to the values at week 0). Data are presented as medians (n = 25 in each group).

The 75 selected participants were randomly assigned to three groups: placebo, postbiotic lozenge, and heat-killed probiotic lozenge. We collected saliva samples at weeks 0, 2, and 4 after oral lozenge intake initiation and measured changes in their microbiota. Plaque weight was 0.37 ± 0.16 g at week 0, and the initial *S. mutans* in saliva (CFUs/mL) was 4.25E+06 ± 2.90E+06. The postbiotic lozenge significantly reduced the oral *S. mutans* bioburden to 60% (median) at week 4 (compared with the postbiotic lozenge at week 0 and placebo at week 4; p < 0.05 for both), and the heat-killed probiotic lozenge significantly reduced *S. mutans* to 61% (median) at week 4 (compared with the probiotic lozenge at week 4, p < 0.05; Figure 2A). Plate Count Agar (PCA) is a common microbiological growth medium used to monitor total viable bacterial populations of a sample [24]. The PCA agar plate was used to analyze the total bacterial population in the oral cavity. The result indicated that administrating postbiotic lozenges significantly decreased the total bacterial load to 98% at week 2 (compared with placebo at week 2, 160%, p < 0.05; Figure 2B) and to 104% at week 4 (compared with placebo at week 4 [187%] and postbiotic lozenge at week 0; p < 0.05 for both).

### Postbiotic and heat-killed probiotic lozenges effectively increased beneficial microbial strains in saliva samples

The change in the *Bifidobacterium* population in the participants’ oral cavity was further measured after oral lozenge intake. The results revealed that the postbiotic oral lozenge significantly increased the *Bifidobacterium* population to 141% at week 4 (compared with the postbiotic lozenge at week 0 and placebo at week 4 [64%], p < 0.05 for both; Figure 2C). Furthermore, the heat-killed probiotic lozenge significantly increased the salivary *Bifidobacterium* population to 111% at week 2 (compared with the probiotic lozenge at week 0 and placebo at week 4, p < 0.05 for both) and to 114% at week 4 (compared with the probiotic lozenge at week 0 [p < 0.05] and placebo at week 4 [p < 0.01]).

The measurement of the *Lactobacillus* population in saliva samples revealed that the postbiotic oral lozenge significantly increased the *Lactobacillus* population to 135% at week 2 (compared with the postbiotic oral lozenge at week 0 [p < 0.05] and placebo at week 2 [p < 0.01]; Figure 2D) and to 227% at week 4 (compared with the postbiotic oral lozenge at week 0 and placebo at week 4; p < 0.001 for both). Furthermore, the heat-killed probiotic lozenge significantly increased the salivary *Lactobacillus* population to 123% at week 2 (compared with the heat-killed probiotic lozenge at week 0 and placebo at week 4; p < 0.05 for both) and to 201% at week 4 (compared with the heat-killed probiotic lozenge at week 0 and placebo at week 4; p < 0.001 for both).

### Postbiotic and heat-killed probiotic lozenges effectively increased IgA concentration in saliva samples

**Figure 3:**
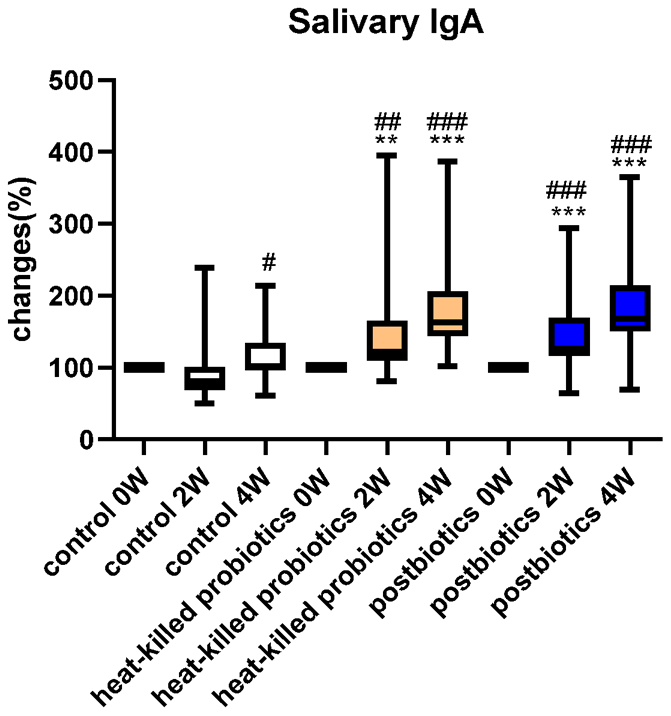
**Oral lozenges significantly increased salivary IgA levels.** Change in *Lactobacillus* (%) in participants’ saliva at 0, 2, and 4 weeks with the intake of oral lozenges. Oral lozenges contained postbiotics or heat-killed cells. Participants in the control group consumed placebo lozenges without the postbiotic content (*p < 0.05, **p < 0.01, and ***p < 0.001 compared with the control group; #p < 0.05, ##p < 0.01, and ###p < 0.001 in reference to values at week 0). Data are presented as medians (n = 25 in each group).

IgA concentration in saliva increased significantly after consuming postbiotic oral lozenges (Figure 3). The postbiotic lozenge significantly increased saliva IgA to 126% at week 2 (compared with the postbiotic lozenge at week 0 [p < 0.05] and placebo at week 2 [p < 0.01]) and to 168% at week 4 (compared with the postbiotic lozenge at week 0 and placebo at week 4; p < 0.001 for both). Moreover, the heat-killed probiotic lozenges significantly increased salivary IgA to 122% at week 2 (compared with the probiotic lozenges at week 0 and placebo at week 2; p < 0.01 for both) and to 163% at week 4 (compared with the probiotic lozenge at week 0 and placebo at week 4; p < 0.001 for both). However, plaque weights did not change much with oral lozenge intake for 4 weeks (Supplementary Figure 2).

### NGS detected oral microbiota changes with oral lozenge intake

We used the NGS technique to analyze microbiota changes in saliva samples with oral lozenge intake. Species heatmap (%) demonstrated that *L. salivarius* significantly increased to 0.03% (compared with the placebo group, p < 0.05) at 4 weeks after consuming postbiotic oral lozenges. Additionally, heat-killed probiotic lozenges significantly increased *L. salivarius* to 0.06% (compared with the placebo group, p < 0.05; Supplementary Figure 3). The result confirmed our previous findings on plate culturing for quantifying *Lactobacillus* in saliva samples (Figure 2D).

**Figure 4:**
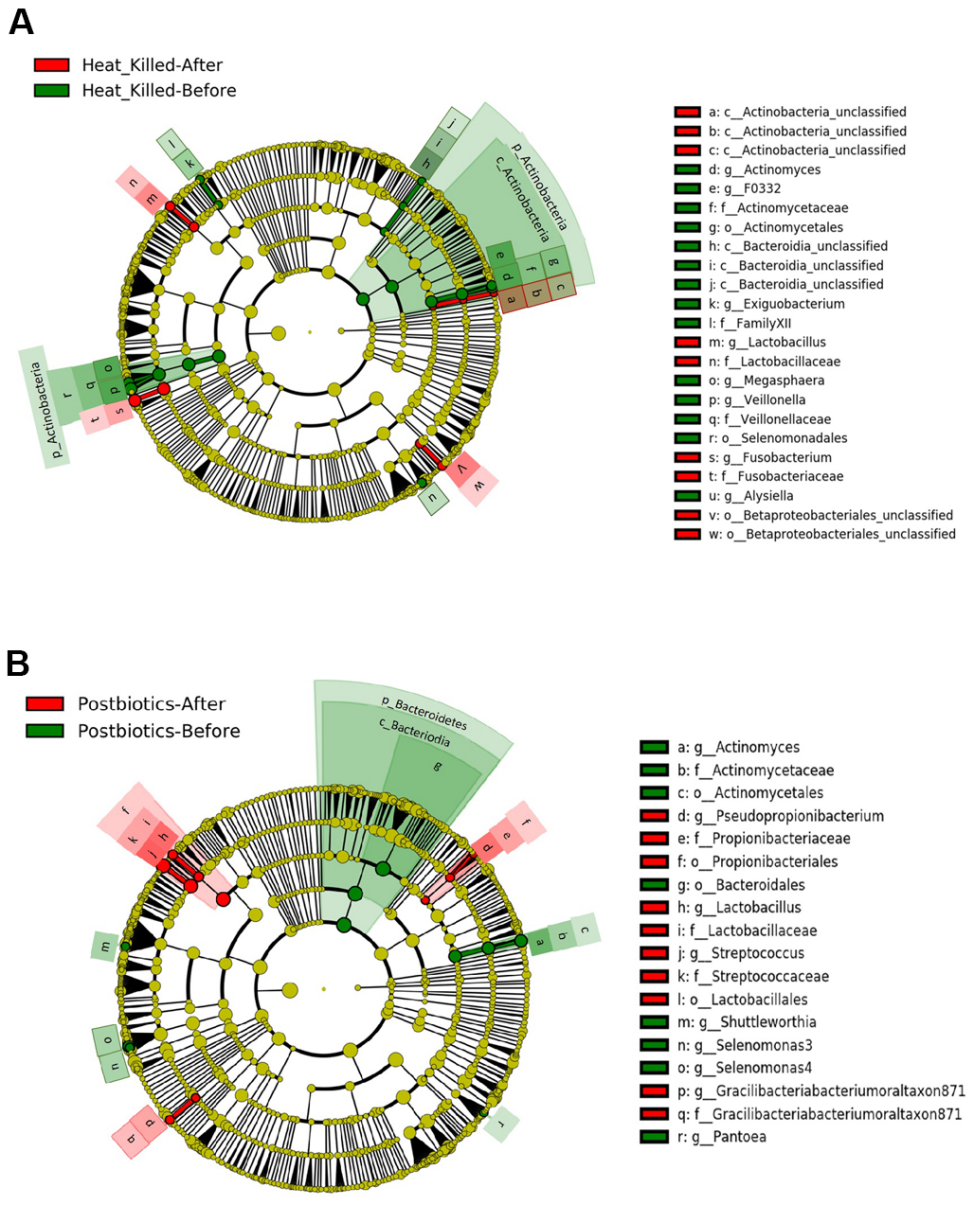
**LEfSe analysis of differential oral microbiota before and after 4 weeks of consuming oral lozenge.** Comparing changes in oral microbiota with the intake of (**A**) heat-killed probiotic lozenges and (**B**) postbiotic lozenges (n = 25 in each group).

LEfSe analysis was used to identify the oral microbiota change between before and after oral lozenge intake. Nine differential bacterial taxa significantly increased after the intake of heat-killed probiotic lozenges, including *Lactobacillus* (Figure 4A). In total, 10 oral bacterial clades significantly increased with the intake of postbiotic lozenges, including *Lactobacillus* (Figure 4B).

**Figure 5:**
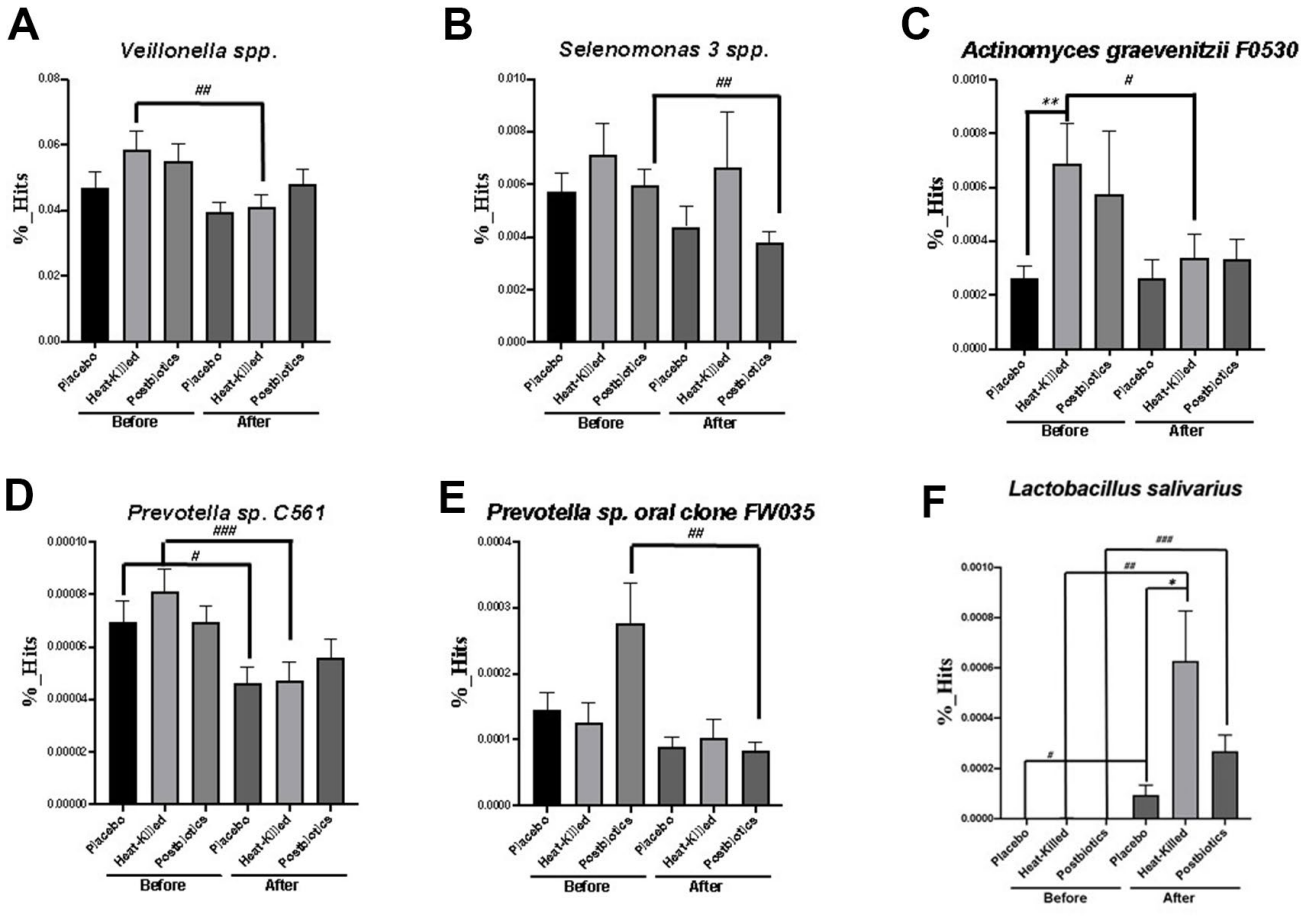
**Significant changes in specific oral bacterial strains after consuming heat-killed probiotic or postbiotic oral lozenges.** Changes in (**A**) *Veillonella* spp., (**B**) *Selenomonas 3* spp., (**C**) *Actinomyces graevenitzii* F0530, (**D**) *Prevotella* sp. C561, (**E**) *Prevotella* sp. oral clone FW035, and (**F**) *L. salivarius* after consuming heat-killed probiotic or postbiotic oral lozenges were analyzed through the LEfSe analysis. Comparing changes in oral bacterial concentration (%) in participants’ saliva at 0 (before) or 4 weeks (after) of oral lozenge intake. The oral lozenges contained postbiotics or heat-killed cells. Participants in the control group consumed placebo lozenges without the postbiotic content (*p < 0.05, **p < 0.01, and ***p < 0.001 compared with the control group; #p < 0.05, ##p < 0.01, and ###p < 0.001 in reference to the values at week 0). Two-tailed t-tests was performed to analyze the statistical difference of experimental results. Data are presented as means ± SDs (n = 25 in each group).

We further analyzed statistical alteration in certain oral bacterial strains with the intake of heat-killed probiotic or postbiotic lozenges (Figure 5A–5F). Pathogenic *Veillonella* spp. (p < 0.01), *Actinomyces graevenitzii* F0530 (p < 0.05), and *Prevotella* sp. C561 (p < 0.001) significantly decreased by 4 weeks of treatment with heat-killed probiotic lozenges. However, the postbiotic oral lozenges significantly reduced the growth of *Selenomonas 3* spp. (p < 0.01) and *Prevotella* sp. oral clone FW035 (p < 0.01). *L. salivarius* significantly increased with the intake of heat-killed probiotic (p < 0.01) and postbiotic (p < 0.001) lozenges.

### Oral lozenges relieved the symptoms of mouth sores, constipation, and gastroesophageal reflux based on health questionnaire analysis

The severity scores for mouth sores or pustule formation decreased to 0.04 ± 0.2 (p < 0.01, compared with the placebo group) and 0.08 ± 0.28 (p < 0.05, compared with the placebo group) after postbiotic lozenge administration for 2 and 4 weeks, respectively (Supplementary Table 1). Furthermore, the heat-killed lozenges relieved the symptoms of mouth sores at weeks 2 (0.23 ± 0.51, p < 0.05, compared with the placebo group) and 4 (0.15 ± 0.37, p < 0.05, compared with the placebo group), respectively. The scores for constipation declined to 0.12 ± 0.33 (p < 0.01) after 4 weeks of postbiotic lozenge intake (Supplementary Table 2). Additionally, 4 weeks after postbiotic oral lozenge intake, the symptoms of gastroesophageal reflux, cold, and drowsiness significantly decreased to 0.12 ± 0.33 (p < 0.05), 0.16 ± 0.37 (p < 0.05), and 0.12 ± 0.33 (p < 0.01), respectively. Moreover, the heat-killed probiotic lozenges diminished the symptoms of constipation, gastroesophageal reflux, cold, and drowsiness at week 4 to 0.15 ± 0.46 (p < 0.05), 0.19 ± 0.4 (p < 0.05), 0.12 ± 0.43 (p < 0.05), and 0.08 ± 0.27 (p < 0.01), respectively.

## DISCUSSION

Based on previous research of viable strain-specific approach [25], we selected three of most appropriate strains to generate oral health promoting products of heat-killed probiotic (ET-66 and AP-32), and postbiotic lozenges (LPL28, ET-66, and AP-32) [22, 23]. Several studies have tested the effect of certain live probiotic strains on oral health. For example, probiotic *Streptococcus salivarius* was reported to reduce severe oral halitosis [26]. However, no study has investigated the role of the postbiotics on regulating oral microbiota and oral immunity. At the beginning of this research, we used *in vitro* antipathogenic assay to demonstrate that individual postbiotics of AP-32, ET-66, and LPL28 can limit the growth rate of oral pathogenic bacteria *S.mutans* and periodontal pathogens *P. gingivalis* BCRC 17689, *P. gingivalis* BCRC 17688, *F. nucleatum* subsp. *polymorphum*, and *A. actinomycetemcomitans* (Figure 1A). Furthermore, the postbiotic lozenge made from mixed metabolites of AP-32, ET-66, and LPL28 exhibited reliable antibacterial function *in vitro* (Figure 1B). The heat-killed probiotic lozenges showed better inhibition rate in *S. mutans* and *A. actinomycetemcomitans* than postbiotic lozenges. Moreover, the heat-killed probiotic lozenges presented excellent bactericidal ability in oral pathogens, which was in accordance with previous findings on individual heat-killed strains [22]. However, the oral lozenges (made from 3 mixed postbiotics) did not inhibit *S. mutans* and *A. actinomycetemcomitans* better than individual ET-66 postbiotic did. This may be because of a lower concentration of functional ingredients in the oral lozenge than in an individual postbiotic (50 mg individual postbiotic versus 50 mg of mixed postbiotics/1 g of lozenge). Besides, previous study revealed viable probiotic lozenges had an excellent inhibition rate (nearly 100%) in five oral pathogenic bacteria *S. mutans* and *P. gingivalis* BCRC 17689, *P. gingivalis* BCRC 17688, *F. nucleatum* subsp. *polymorphum*, and *A. actinomycetemcomitans* [23]. Higher dosage of heat-killed and postbiotic lozenges are presumed to achieve similar pathogenic growth inhibition rate to viable probiotic lozenges. The half maximal inhibitory concentration (IC50) for heat-killed and postbiotic lozenges in limiting oral growth rate should be tested in future.

Next, we validated the antipathogenic ability of heat-killed probiotic or postbiotic lozenges through the detection of changes in the microbial number in saliva.

Compared with the placebo group, the postbiotic and heat-killed lozenge groups exhibited significantly reduced numbers of *S. mutans* and total bacteria at week 4, but no significantly difference at week 2 (Figure 2A, 2B). *S. mutans* is the main pathogen involved in the initiation of dental caries and exhibited a positive correlation with periodontitis [27]. In addition, a high ratio of *S. mutans* DNA was discovered in cardiovascular specimens [28]. Thus, reducing oral *S. mutans* numbers with postbiotic lozenge intake may prevent dental cavity progression, periodontitis, and cardiovascular diseases. Moreover, *Bifidobacterium* and *Lactobacillus* in saliva (cultured on MRS agar plate) revealed that cell numbers increased with the intake of heat-killed probiotic or postbiotic lozenges (Figure 2C, 2D).

Furthermore, postbiotic or heat-killed probiotic lozenges increased IgA concentration in saliva (Figure 3). IgA constitutes 10%–20% of the serum immunoglobulin, second only to IgG. Moreover, IgA present in the mucosal tissues of the oral cavity, digestive tract, and respiratory tract prevents pathogen invasion. Additionally, IgA is present in saliva, tears, and breast milk, particularly in that with high colostrum. No IgA antibody is present in neonatal serum, but newborns obtain IgA secreted from breast milk [29]. Carbohydrate intake may reduce IgA concentration in saliva [30]. Furthermore, salivary IgA acts as the frontline mucosal immune defense against the entry of respiratory pathogens, including severe acute respiratory syndrome coronavirus 2 [31]. Therefore, novel postbiotic or heat-killed probiotic lozenges, which effectively increase salivary IgA concentration, improve bacteriostatic activities, and increase oral populations of beneficial bacteria, may be a potential food product in improving oral health and preventing further infection.

Additionally, we used LEfSe analysis to detect significant changes in oral microbiota after the administration of heat-killed probiotic or postbiotic lozenges (Figure 4). Both heat-killed and postbiotic lozenges showed the ability to significantly increase *Lactobacillus* in the oral cavity. Furthermore, heatmap results of NGS analysis showed an increase in *L. salivarius* level (Supplementary Figure 3). Thus, oral lozenges containing postbiotic or heat-killed probiotic cells promoted the growth and colonization of beneficial microorganism in the oral cavity. In addition, the upregulation of *L. salivarius* in oral microbiota has been reported to promote anticariogenic effects [32, 33]. Moreover, an animal study revealed that *L. salivarius* subsp. *salicinius* AP-32 can eradicate *Helicobacter pylori* infection in addition to improving oral health [34].

Based on previous findings that probiotic strains of AP-32, ET-66 and LPL28 effected oral microbiota [23] and viable strains may improve salivary IgA via up-regulating anti-inflammatory cytokines, IL-10 and TGF-beta [35]. Besides, a previous animal study discovered the mixed viable probiotic strains of *Lactobacillus salivarius* subsp. *salicinius* AP-32, *L. johnsonii* MH-68, *L. reuteri* GL-104, and *Bifidobacterium animalis* subsp. *lactis* CP-9, significantly increased SCFA and MCFA levels. The elevated SCFA and MCFA levels may affect the populations of gut microbiota [36]. The secreted SCFA and MCFA from viable probiotic strains may affect the oral bacterial populations. Some metabolites such as butyrate may stimulate the formation of periodontal/periapical tissues at low or high concentrations [37–39].

However, the detailed mechanism of how three mixed viable probiotic strains (AP-32, ET-66, and LPL28) altered the oral microbiome should be tested in the future. Here, we further discovered that postbiotic would also improve oral microbiota. Nevertheless, the clinical oral health improving function should be tested after stopping consuming postbiotic lozenges. A larger scale clinical analysis of oral microbiota and metabolite profiling for the development of personalized oral therapy in the future [40].

The heat-killed lozenges were efficacious in reducing growth of pathogenic *Veillonella* spp., *A. graevenitzii* F0530, and *Prevotella* sp. C561. Moreover, the postbiotic lozenges reduced the growth of *Selenomonas* 3 spp. and *Prevotella* sp. oral clone FW035 (Figure 5). *Veillonella* spp. has been reported to be associated with halitosis [41] and dental caries [42]. The overgrowth of *Prevotella* spp. may lead to halitosis [41] and periodontal disease [43]. *A. graevenitzii* has been discovered to cause pulmonary abscess [44], pneumonia [45], and dental caries [42]. Thus, heat-killed probiotic lozenges might improve the oral smell and oral hygiene by reducing the oral population of *Veillonella* spp., *Prevotella* spp., and *A. graevenitzii*. Additionally, postbiotic oral lozenges may significantly reduce *Selenomonas* spp. and *Prevotella* spp., which are associated with halitosis [41]. Moreover, the oral health questionnaires presented that heat-killed and postbiotic lozenges would significantly improve symptoms of ruptured mouth, drool (Supplementary Table 1), constipation, gastroesophageal reflux, cold, drowsiness (Supplementary Table 2). The results of questionnaires at present study are in accordance with previous findings in viable probiotic lozenges. The questionnaires for viable probiotic lozenge present additional improvements in teeth bleeding, sore throat, and stomach pain [23].

Finally, Ishikawa, K. H. et al. demonstrated postbiotics would effectively limit the formation of biofilm formation and growth rate of *A. actinomycetemcomitans* [21]*.* At present study, it demonstrated that postbiotic significantly reduced the survival rate of other oral pathogens including *S. mutans* and periodontal pathogens *P. gingivalis* BCRC 17689, *P. gingivalis* BCRC 17688, *F. nucleatum* subsp. *polymorphum*. We also measured changes in participants’ salivary IgA and oral microbiota by consuming lozenges of postbiotic AP-32 (*L. salivarius* subsp. *salicinius*), ET-66 (*L. paracasei*), and LPL28 (*L. plantarum*). The different mixing proportion of three postbiotics effected on oral hygiene should be tested in future.

In conclusion, the postbiotics and heat-killed probiotics have the advantages of preservation and stable viability over viable strains. Here, we found that lozenges of postbiotic AP-32 (*L. salivarius* subsp. *salicinius*), ET-66 (*L. paracasei*), and LPL28 (*L. plantarum*) and the heat-killed probiotics of AP-32 (*L. salivarius* subsp. *salicinius*) and ET-66 (*L. paracasei*) were beneficial to oral health. Previous study demonstrated that three strains had excellent antimicrobial activity in zone of inhibition test. The present clinical study revealed that postbiotic or heat-killed probiotic lozenges could effectively reduce the number of *S. mutans* in the oral cavity, increase *L. salivarius* in oral microbial flora, increase salivary IgA concentration, and decrease oral infections. Furthermore, results from the subjective questionnaire revealed that improved oral health was associated with attenuated intestinal symptoms, relieved constipation, and reduced gastroesophageal reflux, stomach pain, colds, and sense of drowsiness. This study suggested that deactivated probiotic cells and their postbiotics can serve as supporters to optimize the efficacy of oral health supplements. However, further experiments are required.

According to the current manufacturing regulations of cosmetics and cleaning products in various countries, the inactive substances of functional lactic acid bacteria are more suitable for the industrial application. Therefore, this study presented potential food-grade supplementations for promoting oral health and applicable food industrial products in the future.

## MATERIALS AND METHODS

### Oral lozenges of heat-killed probiotics and postbiotics

Three probiotic strains known for their antipathogenic against oral pathogens, namely *L. salivarius* subsp. *salicinius* AP-32, *L. paracasei* ET-66, and *L. plantarum* LPL28, were obtained from Bioflag biotech. Co. Ltd (Tainan, Taiwan). *L. salivarius* subsp. *salicinius* AP-32 was isolated from healthy human intestine and deposited in Food Industry Research and Development Institute, Taiwan (ID: BCRC 910437) and in Wuhan university, China (ID: CCTCC-M2011127); *L. paracasei* ET-66 was isolated from healthy human breast milk and deposited in Food Industry Research and Development Institute, Taiwan (ID: BCRC 910753) and in China General Microbiological Culture Collection Center, Beijing, China (ID: CGMCC-13514). *L. plantarum* LPL28 was isolated from fermented food Mizo and deposited in Food Industry Research and Development Institute, Taiwan (ID: BCRC 910536) and in China General Microbiological Culture Collection Center, Beijing, China (ID: CGMCC-17954).

We collected postbiotics from these three strains (50 mg of mixed postbiotics/1 g of lozenge) to develop oral lozenges [46]. The detailed procedure for producing postbiotic powder is described as follow: Incubating three probiotic strains AP-32, ET-66 and LPL28 (2 × 10^11^ colony-formation units [CFUs]/g) in De Man, Rogosa and Sharpe (MRS) media (Difco. Laboratories, Detroit, MI, USA) at 37° C for 48 h to obtain viable probiotics strains. Fermenting mixed probiotics stains of AP-32, ET-66 and LPL28 (the concentration of each probiotic strain was 1 × 10^9^ CFU/mL) with nitrogen sources (skimmed milk and soy bean) and carbohydrate sources (glucose, fructose) at 37° C for 16 hours. Collecting fermented supernatant (postbiotic solution) after centrifugation at 15,000 x g. Pasteurizing fermented product with ultra-high-temperature (UHT) to 135–140° C for 4 seconds. Then spray-drying fermented solution into postbiotic powder. The major nutritional components of postbiotic powder (per 100 g contribution) were crude protein 15.9 g, crude fat 1.9 g, saturated fat 0.21 g, carbohydrate 65.3 g, sugar 3.799 g, glucose 0.553 g, sucrose 0.082 g, maltose 0.667 g, lactose 2.497 g, sodium 3062.7 mg, and calories 341.9 Kcal.

We incubated two probiotic strains *L. salivarius* subsp. *salicinius* AP-32 and *L. paracasei* ET-66 (2 × 10^11^ colony-formation units [CFUs]/g) in De Man, Rogosa and Sharpe (MRS) media (Difco. Laboratories, Detroit, MI, USA) at 37° C for 48 h to obtain viable probiotics strains [22]. Fermentation and centrifugation procedure was the same as making postbiotic product. Collecting and pasteurizing pellet with ultra-high-temperature (UHT) to 135–140° C for 4 seconds. Freeze-drying the pasteurized pellet for 40 hr, and then obtaining heat-killed probiotic powder. The heat-killed probiotic oral lozenges were composed of 10^10^ CFUs/g of cells. Furthermore, food-grade D-sorbitol, erythritol, fructooligosaccharides, lactose, magnesium stearate, silica, and sucralose were used to prepare placebo oral lozenges. Furthermore, food-grade D-sorbitol, erythritol, fructooligosaccharides, lactose, magnesium stearate, silica, and sucralose were used to prepare placebo oral lozenges.

### Oral pathogenic bacteria

We used tryptic soy broth (TSB; Merck KGaA, Darmstadt, Germany) supplemented with 5% sheep’s blood to cultivate *P. gingivalis* and *F. nucleatum* subsp. *polymorphum* and brain heart infusion (BHI; Merck KGaA, Darmstadt, Germany) broth for culturing *A. actinomycetemcomitans*. Additionally, TSB was used to cultivate *Streptococcus mutans*. We incubated pathogens at 37° C (48 h) for subsequent antibacterial tests. *S. mutans* BCRC 10793T, *P. gingivalis* BCRC 17689, *P. gingivalis* BCRC 17688, *F. nucleatum* subsp. *polymorphum* BCRC 17679, and *A. actinomycetemcomitans* BCRC 14405 were obtained from Bioresource Collection and Research Center (BCRC), Hsinchu, Taiwan.

### Analyzing bacteriostatic activities

The three probiotic strains were individually cultured in MRS media at 37° C for 20 h. Then, 4.9 mL of supernatants were collected and mixed with oral pathogenic bacteria (10^6^ CFUs/0.1 mL) after which the mixed solution was incubated at 37° C for 48 h. Subsequently, the CFUs of pathogenic bacteria in each tube were calculated. Furthermore, the CFUs of oral pathogens were compared with the control media, which contained pathogens without postbiotic treatment.

The bacteriostatic activities of postbiotic and heat-killed probiotic lozenges were tested according to the same protocol. The experimental lozenges were dissolved in either a TSB or BHI medium at 0.1 g/mL concentration, and then, oral pathogens (10^6^ CFU) were introduced into the lozenge solutions and coincubated at 37° C for 2 (*S. mutans*) or 3 (*P. gingivalis*, *F. nucleatum* subsp. *polymorphum*, and *A. actinomycetemcomitans*) days. Furthermore, the CFUs of pathogenic bacteria in each tube were calculated. We measured the survival rates of the oral pathogens by using the following formula: CFU_experimental group_/CFU_control media_ (%). The inhibition rates of the oral pathogens were determined using the following formula: 1 − survival rate (%). The metabolites of *L. rhamnosus* GG (LGG) purchased from Chr. Hansen, Hoersholm, Denmark were tested as positive control.

### Participants

In total, 75 participants (on-smokers, free from systemic diseases) between 20 and 40 years of age and with *S. mutans* >10^5^ CFUs/mL in their saliva samples were recruited. Their average age was 26.29 ± 5.59 years. The initial amount of *S. mutans* in their saliva (CFUs/mL) was 4.25* 10^6^ ± 2.90* 10^6^, and their plaque weight was 0.37 ± 0.16 g. All clinical tests were performed according to the guidelines of the Ministry of Health and Welfare, Taiwan (Health Food Evaluation No. 88037803). All participants were randomly and blindly assigned to three groups: placebo, heat-killed probiotics, and postbiotics (25 participants in each group). Participants were asked to clean their oral cavity and then consume three oral lozenges (3 g) every day for 4 weeks [47]. We collected and measured oral microbiota, IgA levels, and oral pathogens in 2-mL saliva samples at weeks 0, 2, and 4. Additionally, total plaque and oral health questionnaire were analyzed at weeks 0, 2, and 4. The protocols for evaluating the uptake of postbiotic products, colleting human saliva samples, and administering subjective questionnaires were approved by the Institutional Review Board of Chung Shan Medical University, Taiwan (CS19052).

### Analysis of the populations of *Lactobacillus*, *Bifidobacterium*, *S. mutans*, and total aerobic bacteria in the oral cavity

For analyzing the populations of *Lactobacillus*, *Bifidobacterium*, total aerobic bacteria, and *S. mutans* in the oral cavity, 100-μL saliva samples were cultured on MRS with 0.05% cysteine agar, plate count agar (PCA; Merck KGaA, Darmstadt, Germany), and mitis salivarius-bacitracin (MSB Agar) (Merck KGaA, Darmstadt, Germany) in triplicate, and CFUs on each plate were calculated. The change rates of oral pathogen were determined using the following formula: (CFUs_week 2 or 4_ − CFUs_week 0_)/CFUs_week 0_ (%).

### Next-generation sequencing analysis of oral microbiota

Changes in oral microbiota were measured using the next-generation sequencing (NGS) technique. Microbial DNA was extracted from the saliva samples and sent to Genomics Co. Ltd. for NGS analysis. In brief, commercial specific primers (Genomics Co. Ltd., Taiwan) were used to amplify the amplicon DNA segments (16S rRNA and 16S V3–V4) by using the polymerase chain reaction (PCR) technique (Phusion High-Fidelity PCR Master Mix, New England Biolabs, USA). The PCR products of 400–450 bp were purified using the Qiagen Gel Extraction kit (Qiagen, Germany). Then, the TruSeq DNA PCR-free sample preparation kit (Illumina, USA) was used to generate sequencing libraries with provided index codes. The Qubit 2.0 Fluorometer (Thermo Scientific, USA) and Agilent Bioanalyzer 2100 system (Agilent Technologies, Inc., USA) were applied to confirm the library quality. Finally, the Illumina HiSeq 2500 platform was used to sequence and analyze the DNA library. QIIME software, version 1.7.0, was used to analyze NGS raw data [48]. The sizes of each single taxon of the groups were further analyzed through linear regression plots and linear discriminant analysis effect size (LEfSe) analysis (https://huttenhower.sph.harvard.edu/galaxy/) and the analysis protocol was followed the instruction in https://twbattaglia.gitbooks.io/introduction-to-qiime/content/lefse.html.

### Measuring IgA and plaque

The human IgA enzyme-linked immunosorbent assay (ELISA) kit (Invitrogen, Lot: 218315-003) was used to measure IgA concentrations of saliva samples in triplicate. The IgA concentration was analyzed at an optical density of 450–570 nm by using the ELISA reader. Plaque in the mouth was collected using a swab. Then, the dehydrated plaque was weighed. The weight of plague (g) = total weight of samples with sample tubes (g) – sample tubes (g).

### Questionnaire of dental problems and gastrointestinal symptoms

Self-report questionnaires were used to evaluate common dental symptoms and a gastrointestinal symptom [23]. All participants completed the questionnaire at weeks 0, 2, and 4 after the intervention. Participants could give the following responses: 0 = no symptom; 1 = mild; 2 = medium; 3 = serious.

### Statistics

GraphPad Prism software (San Diego, CA, USA) was applied to perform statistical analysis of collected data. Data of bacterial colonies in oral and salivary IgA are presented as medians. Each test was performed triplicate. The rest of the data are presented as means ± standard deviations (SDs) or means. Two-tailed t-tests were performed to analyze the statistical differences in experimental results. Statistical difference was indicated by p < 0.05. A significant statistical difference was observed in the treatment data of the experimental groups compared with their pretreatment data at week 0 (# p < 0.05) and compared with the placebo group data at week 4 (* p < 0.05).

## Supplementary Material

Supplementary Figures

Supplementary Tables
