# Supplementary material for: Impact of the food grade heat-killed probiotic and postbiotic oral lozenges in oral hygiene
Source: Aging (Albany NY). 2022 Mar 2;14(5):2221–38. doi: 10.18632/aging.203923 (PMC8954981; doi:10.18632/aging.203923)
Supplement: Supplementary Figures [file aging-14-203923-s001.pdf]

SUPPLEMENTARY FIGURES

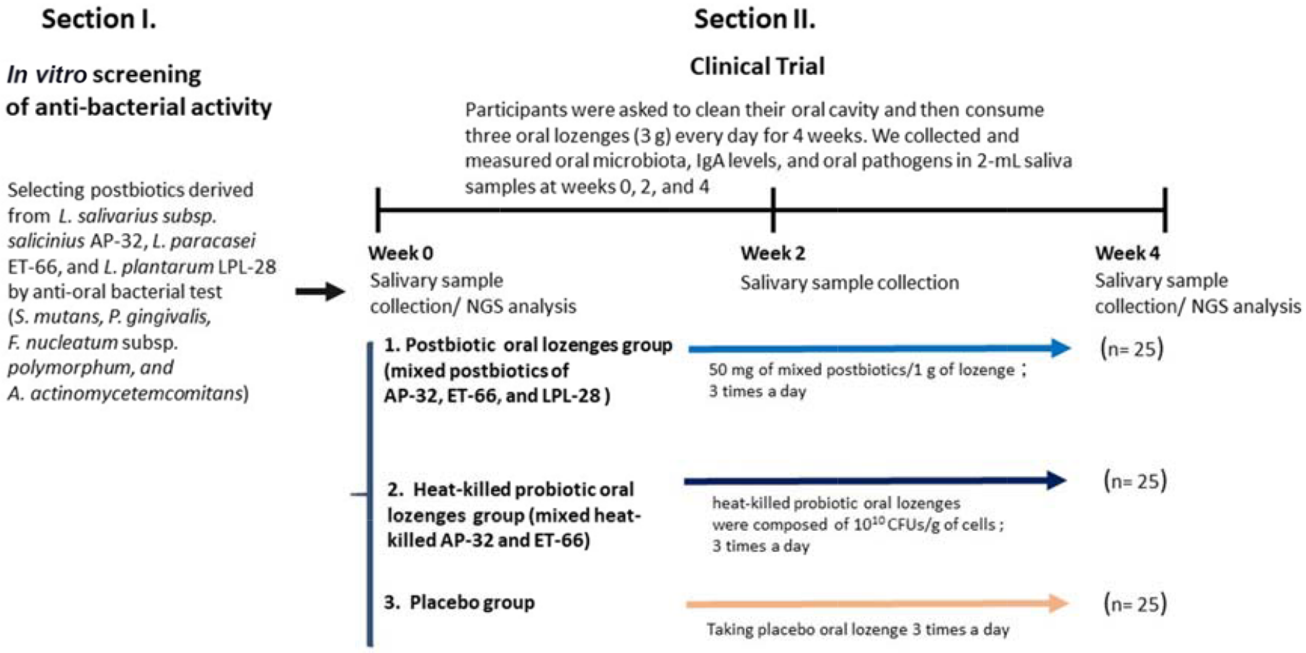

Supplementary Figure 1. Experimental design.

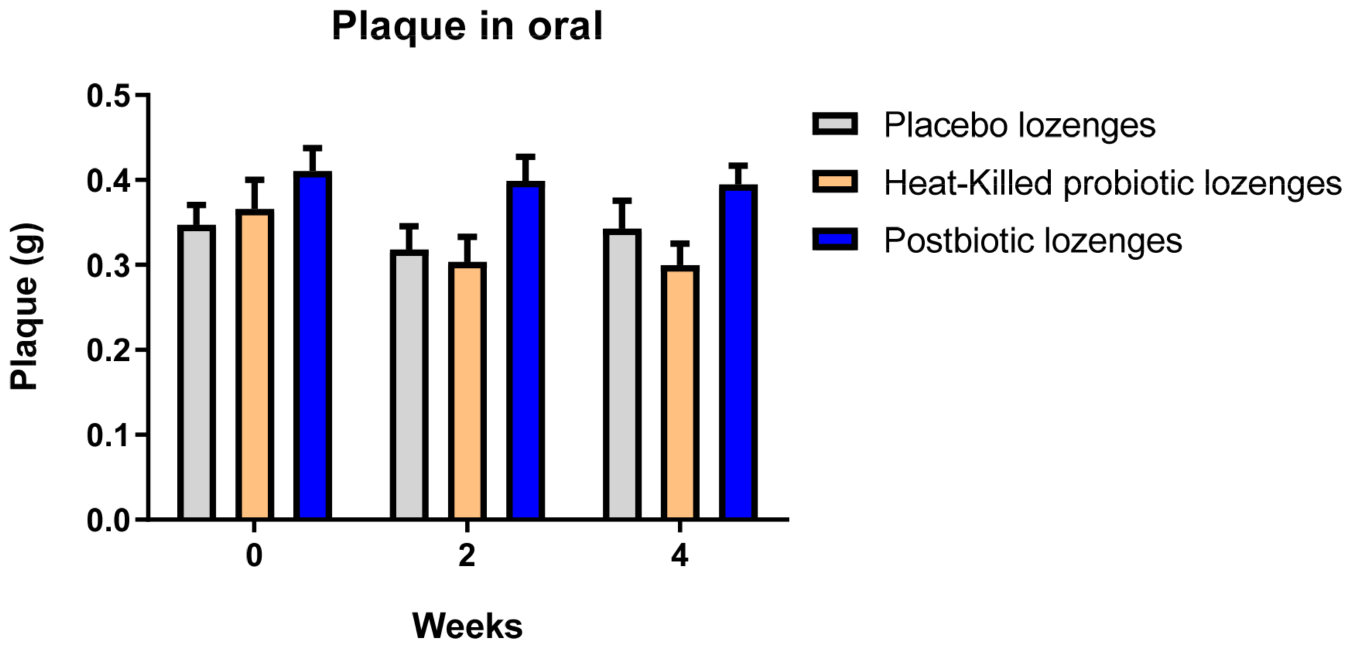

Supplementary Figure 2. Plaque weight analyses. The oral lozenges contained postbiotics or heat-killed cells. Participants in the control group consumed placebo lozenges without the postbiotic content (no significant difference was observed between the groups). Data are presented as mean ± SD.

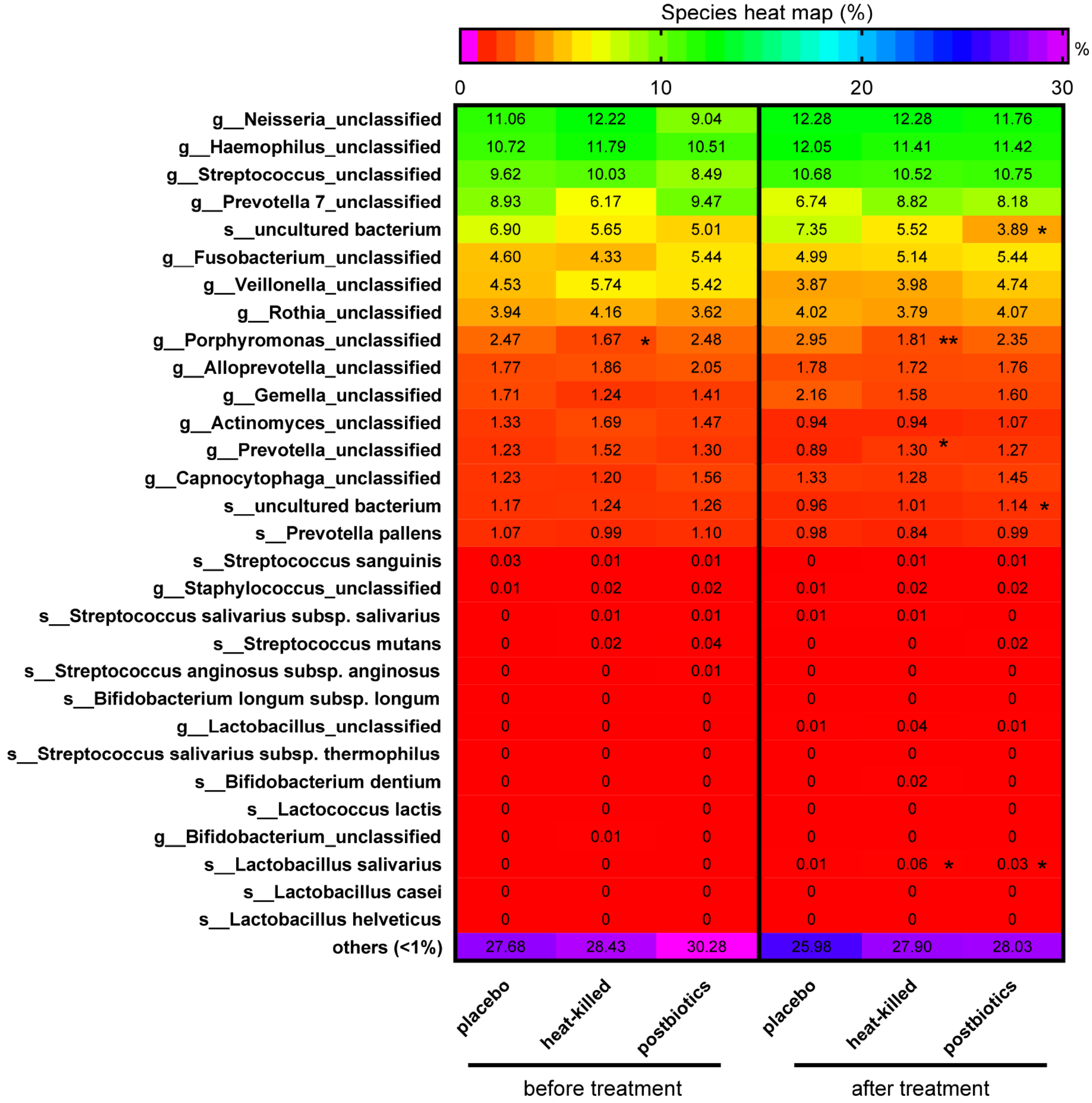

Supplementary Figure 3. NGS for detecting changes in oral microbiota with the intervention of oral lozenges. Heatmap presents oral microbiota changes (genus, %) in participants' saliva before and after 4 weeks of consuming oral lozenge. Participants in the control group consumed placebo lozenges without the postbiotic content (*p < 0.05 and **p < 0.01 indicate significant change compared with the placebo group). Data are presented as means.
