# Supplementary material for: Impact of the food grade heat-killed probiotic and postbiotic oral lozenges in oral hygiene
Source: Aging (Albany NY). 2022 Mar 2;14(5):2221–38. doi: 10.18632/aging.203923 (PMC8954981; doi:10.18632/aging.203923)
Supplement: Supplementary Tables [file aging-14-203923-s002.pdf]

SUPPLEMENTARY TABLES

Supplementary Table 1. Analyses of the oral health questionnaire.

0W		Placebo		Heat-killed probiotics			Postbiotics		
		2W	4W	0W	2W	4W	0W	2W	4W
Toothache / Gingival Swelling[c]	0.4±0.71	0.2±0.41	0.16±0.47	0.31±0.68	0.19±0.49	0.19±0.69	0.48±0.59	0.28±0.54	0.16±0.37
Teeth bleeding, while brushing[c]	0.48±0.78	0.44±0.65	0.44±0.65	0.46±0.81	0.27±0.53	0.31±0.62	0.44±0.58	0.28±0.61	0.16±0.37
Ruptured mouth or pustule formation[c]	0.6±0.76	0.72±0.98	0.52±0.82	0.5±0.71	0.23±0.51[a]	0.15±0.37[a]	0.44±0.82	0.04±0.2[b]	0.08±0.28[a]
Sore throat[c]	0.48±0.59	0.4±0.87	0.36±0.76	0.42±0.7	0.19±0.49	0.19±0.4	0.28±0.46	0.2±0.41	0.16±0.47
Drool[c]	0.32±0.56	0.52±0.82	0.48±0.65	0.38±0.57	0.12±0.33[a]	0.15±0.46[a]	0.6±0.76	0.12±0.33[a]	0.16±0.37[a]
Cough[c]	0.32±0.48	0.24±0.6	0.4±0.76	0.62±0.8	0.27±0.6	0.54±0.81	0.36±0.57	0.12±0.44	0.12±0.44

[a]Significant difference compared with the placebo group, $p < 0.05$.
[b]Highly significant difference compared with the placebo group, $p < 0.01$.
[c]Scores were evaluated according to severity: 3 points = severe; 2 points = normal; 1 point = mild; 0 point = none; the data are presented as mean ± SD collected from all groups.

Supplementary Table 2. Intestinal symptoms based on health survey evaluation[c].

0W		Placebo		Heat-killed probiotics			Postbiotics		
		2W	4W	0W	2W	4W	0W	2W	4W
Defecation[d]	0.08±0.28	0.2±0.41	0.36±0.49	0.23±0.43	0.38±0.5	0.46±0.58	0.2±0.41	0.4±0.5	0.32±0.48
Constipation[c]	0.48±0.65	0.44±0.65	0.6±0.71	0.31±0.68	0.15±0.46	0.15±0.46[a]	0.28±0.46	0.16±0.37	0.12±0.33[b]
Diarrhea[c]	0.2±0.41	0.32±0.63	0.36±0.64	0.27±0.53	0.15±0.46	0.15±0.46	0.12±0.33	0.24±0.66	0.16±0.37
Stomach pain[c]	0.68±0.8	0.4±0.65	0.32±0.63	0.35±0.49	0.23±0.43	0.23±0.43	0.36±0.49	0.12±0.33	0.16±0.37
Gastroesophageal[c] Reflux[c]	0.68±0.9	0.48±0.65	0.52±0.71	0.31±0.47	0.15±0.37[a]	0.19±0.4[a]	0.28±0.46	0.08±0.28[b]	0.12±0.33[a]
Cold[c]	0.36±0.57	0.44±0.71	0.56±0.82	0.46±0.58	0.23±0.51	0.12±0.43[a]	0.32±0.47	0.2±0.41	0.16±0.37[a]
Drowsiness[c]	0.36±0.7	0.4±0.58	0.52±0.65	0.31±0.47	0.15±0.37	0.08±0.27[b]	0.32±0.56	0.16±0.37	0.12±0.33[b]

[a]Significant difference compared with the placebo group, $p < 0.05$.
[b]Highly significant difference compared with the placebo group, $p < 0.01$.
[c]Scores were evaluated according to severity: 3 points = severe; 2 points = normal; 1 point = mild; 0 point = none; the data are presented as mean ± SD collected from all groups.
[d]Scores were analyzed based on frequency: >3 days = 2 points; once every 3 days = 1 point; more than once a day = 0 points.
